# Acute toxic exposures in children: analysis of a three year registry managed by a Pediatric poison control Center in Italy

**DOI:** 10.1186/s13052-021-01071-y

**Published:** 2021-06-02

**Authors:** M. Marano, F. Rossi, L. Ravà, M. Khalil Ramla, M. Pisani, G. Bottari, L. Genuini, G. Zampini, J. Nunziata, A. Reale, M. A. Barbieri, F. Celeani, M. Di Nardo, C. Cecchetti, F. Stoppa, A. Villani, M. Raponi, S. Livadiotti, G. Pontrelli

**Affiliations:** 1 DEA Paediatric Intensive Care Unit, Children’s Hospital Bambino Gesù, IRCCS, Rome, Italy; 2 Paediatric Clinical Toxicology Centre, Children’s Hospital Bambino Gesù, IRCCS, Rome, Italy; 3 Emergency Department, Children’s Hospital Bambino Gesù, IRCCS, Palidoro, Italy; 4 Epidemiology Unit and Biostatistics, Children’s Hospital Bambino Gesù, IRCCS, Rome, Italy; 5 Emergency Department, Children’s Hospital Bambino Gesù, IRCCS, Rome, Italy; 6 Information Systems Department, General direction Children’s Hospital Bambino Gesù, IRCCS, Rome, Italy; 7 Medical Direction, Children’s Hospital Bambino Gesù, IRCCS, Rome, Italy; 8 Clinical Trials Centre, University Department of Paediatrics, Bambino Gesù Children’s Hospital, IRCCS, Rome, Italy

**Keywords:** Paediatric poisoning, Intoxication, Pharmaceuticals, Emergency department, Childhood, Poison control Centre

## Abstract

**Introduction:**

Acute pediatric poisoning is an emerging health and social problem. The aim of this study is to describe the characteristics of a large pediatric cohort exposed to xenobiotics, through the analysis of a Pediatric Poison Control Center (PPCc) registry.

**Methods:**

This study, conducted in the Pediatric Hospital Bambino Gesù of Rome, a reference National Pediatric Hospital, collected data of children whose parents or caregivers contacted the PPCc by phone (group “P”), or who presented to the Emergency Department (group “ED”), during the three-year period 2014–2016. Data were prospectively and systematically collected in a pre-set electronic registry. Comparisons among age groups were performed and multivariable logistic regression models used to investigate associations with outcomes (hospital referral for “P”, and hospital admission for “ED”group).

**Results:**

We collected data of 1611 children on group P and 1075 on group ED. Both groups were exposed to both pharmaceutical and non-pharmaceutical agents. Pharmaceutical agent exposure increased with age and the most common route of exposure was oral. Only 10% among P group were symptomatic children, with gastrointestinal symptoms. Among the ED patients, 30% were symptomatic children mostly with gastrointestinal (55.4%) and neurologic symptoms (23.8%). Intentional exposure (abuse substance and suicide attempt), which involved 7.7% of patients, was associated with older age and Hospital admission.

**Conclusions:**

Our study describes the characteristics of xenobiotics exposures in different paediatric age groups, highlighting the impact of both pharmacological and intentional exposure. Furthermore, our study shows the utility of a specific PPCc, either through Phone support or by direct access to ED. PPCc phone counselling could avoid unnecessary access to the ED, a relevant achievement, particularly in the time of a pandemic.

## Background

Poisoning is a common and potentially life-threatening public health problem, which contributes to the added costs of both emergency and in-patient care. Although poisoning may occur at any age, children are at particular risk. According to the American Association of Poison Control Centres (AAPCC), about 59% of poisoning cases in the United States involved patients under the age of 20 [1]. As far as we know, this is one of the few dedicated reports describing a large cohort of children exposed to xenobiotics in Europe and stratified by age groups. Improving our understanding of the current problem of paediatric poisoning may provide valuable informations for future education efforts, prevention strategies, public policies and treatment decisions. The aim of this study was to analyze the characteristics of a paediatric population exposed to xenobiotics, recorded over a three-year period by the Paediatric Poison Control Centre (PPCc) of the National referenced Children Hospital in Italy. The PPCc provides support to families of poisoned paediatric patients requiring counselling through an active phone line, which is accessible by parents and primary care/hospital physicians, or by providing direct access to the Emergency Department.

### Study design, population and setting

In this observational study, we prospectively collected data of children, aged between 0 and 18 years, whose parents or primary care personnel contacted the PPCc by phone call (PPCcP) after poison exposure or who presented to the Emergency Department of the Paediatric Hospital “Bambino Gesù” of Rome from January, 1st of 2014 to December, 31st of 2016.

We divided children in two different populations: “P” group, cases for whom medical advice was asked by phone and “ED” group, cases who arrived directly to the Emergency Department. Some cases arrived to the ED after a phone call (group “PED”) and therefore were considered in both groups for the analyses.

### Measurements

Data were registered in a structured database including demographic and clinical data as: gender; age, (which was further categorised into the following groups): infant (0–11 months old), pre-school (1–5 years old), school (6–11 years old), adolescents (12–18 years old); time of exposure (morning, afternoon, evening, night); location of poison exposure (home or outside); reason for poison exposure (accidental or intentional). Accidental exposure was also categorized as medication error (due to caregiver mistake, without symptoms) and adverse drug reaction (ADR), while intentional exposure was distinguished into substance abuse and suicide attempt (defined by the will to commit suicide).

Substances taken by patients where categorized in two main groups, *pharmaceutical* and *not-pharmaceutical* substances. The first included analgesics (including anti-inflammatories, opioid and antispasmodics), antimicrobials (antibacterials, antivirals and antifungals), antihistamines, cardioactives, hormones and gastrointestinal drugs, supplements, vitamins, neuroactive (including barbiturates, benzodiazepines, neuroleptics, antidepressant, antiepileptic drugs), respiratory, topical, homeopathic, others. *Non-pharmaceutical* substances included cosmetics (including all products for personal care such as shampoos, bath soaps, body lotions), domestic products category (including household cleaners, laundry pods, dishwasher tablets), industrial products (including petrol derived products, essential oils, disk batteries, glues, inks, plant fertilizers, degreasers, antifreeze, foreign bodies and various other miscellaneous products), caustics, foods, pesticides (usually used at home against parasites and insects), plants/mushrooms, abuse substances (including alcohol and recreational drugs), viper bites, botulinum, carbon monoxide/fumes. Route of exposure included oral, cutaneous, inhalation, mucosal, ocular, bite, injection, other. Clinical presentations were divided in *asymptomatic* or *symptomatic* (dermatological, gastrointestinal, neurological, respiratory, cardiovascular, multisystemic, other). Finally, treatments used for decontamination were recorded and included charcoal, gastric lavage, ocular irrigation, topic wash.

### Outcomes

In group P severity was defined by the need for hospital referral for clinical investigation whilst in group ED severity criteria were defined by need for hospitalization, length of hospitalization, admission to the Paediatric Intensive Care Unit (PICU) and death.

### Statistical analysis

Descriptive analysis for group P and group ED was reported and stratified according to age group. Categorical data was presented as count and proportions and continuous data were presented as mean with standard deviation or median with interquartile range. Comparisons among proportions were performed through Chi-square test or Fisher exact test as appropriate.

Multivariable logistic regression models were used in order to investigate associations among outcomes (hospital referral for population “P”, and hospital admission for population “ED”) and possible covariates while adjusting for confounders. Results with a *p*-value < 0.05 were considered statistically significant. Statistical analysis were performed by using Stata 15.0 (StataCorp).

## Results

### Children characteristics

We prospectively collected data of 2566 children aged 0–18, divided into the following groups:

- Group P: 1611 children whose parents or caregivers needed phone counselling by contacting the PPCcP, among these 120 patients were additionally visited in our ED (Subgroup PED, also part of group “ED”).
- Group ED: 1075 children brought to our hospital. The total number of children admitted to our ED over the described period was 237.323, hence children admitted for exposure to xenobiotics were 0.45%.

### Group P

In this population, described in Tables 1, 51.5% of children were males; prevalent age was pre-school (74.1%), afternoon and evening (74.7%) were the most frequent times of exposure; the most frequent location was at home (95.6%).

**Table 1:** Characteristics of Group “P”, cases for whom PPCc was contacted by phone

Age Category	Infant n (%)	Pre-School n (%)	School n (%)	Adolescents n (%)	Total n (%)	***p***-value
**Gender**						
Male	111 (46.8)	623 (52.2)	77 (55.4)	18 (42.9)	829 (51.5)	0.220
Female	126 (53.2)	570 (47.8)	62 (44.6)	24 (57.1)	782 (48.5)	
**Time of exposure**						
Morning	73 (30.8)	252 (21.1)	35 (25.2)	11 (26.2)	371 (23.0)	**< 0.001**
Afternoon	85 (35.9)	442 (37.0)	33 (23.7)	15 (35.7)	575 (35.7)	
Evening	78 (32.9)	476 (39.9)	62 (44.6)	12 (28.6)	628 (39.0)	
Night	1 (0.4)	23 (1.9)	9 (6.5)	4 (9.5)	37 (2.3)	
**Location of poison exposure**						
Home	221 (93.6)	1148 (96.2)	129 (92.8)	41 (97.6)	1539 (95.6)	0.101
Outside	15 (6.4)	45 (3.8)	10 (7.2)	1 (2.4)	71 (4.4)	
**Reason for poison exposure**						
**Accidental exposure**	**237 (100.0)**	**1193 (100.0)**	**127 (98.6)**	**32 (76.2)**	**1599 (98.2)**	**< 0.001**
*General accidental exposure*	199 (84.0)	1116 (93.5)	111 (79.9)	26 (61.9)	1452 (90.1)	
*Medication error*	37 (15.6)	76 (6.4)	24 (17.3)	5 (11.9)	142 (8.8)	
*Adverse reaction*	1 (0.4)	1 (0.1)	2 (1.4)	1 (2.4)	5 (0.3)	
**Intentional exposure**	**0 (0.0)**	**0 (0.0)**	**2 (1.4)**	**10 (23.8)**	**12 (0.8)**	
*Suicide attempt*	0 (0.0)	0 (0.0)	2 (1.4)	9 (21.4)	11 (0.7)	
*Abuse*	0 (0.0)	0 (0.0)	0 (0.0)	1 (2.4)	1 (0.1)	
**Categories of exposure**						
**Pharmaceutical**	**75 (31.6)**	**383 (32.1)**	**58 (41.7)**	**20 (47.6)**	**536 (33.3)**	**< 0.001**
*Analgesic*	17 (7.2)	87 (7.3)	9 (6.5)	3 (7.1)	116 (7.2)	
*Hormones*	5 (2.1)	53 (4.4)	4 (2.9)	3 (7.1)	65 (4.0)	
*Antimicrobial*	13 (5.5)	35 (2.9)	5 (3.6)	1 (2.4)	54 (3.4)	
*Topic-Dermatological*	13 (5.5)	36 (3.0)	2 (1.4)	3 (7.1)	54 (3.4)	
*Respiratory*	4 (1.7)	42 (3.5)	4 (2.9)	0 (0.0)	50 (3.1)	
*Neuroactive*	2 (0.8)	30 (2.5)	7 (5.0)	6 (14.3)	45 (2.8)	
*Cardioactive*	5 (2.1)	32 (2.7)	5 (3.6)	1 (2.4)	43 (2.7)	
*Antistaminic*	4 (1.7)	16 (1.3)	7 (5.0)	1 (2.4)	28 (1.7)	
*Supplements*	3 (1.3)	17 (1.4)	3 (2.2)	1 (2.4)	24 (1.5)	
*Gastrointestinal*	5 (2.1)	14 (1.2)	2 (1.4)	1 (2.4)	22 (1.4)	
*Other*	3 (1.3)	12 (1.0)	7 (5.0)	0 (0.0)	22 (1.4)	
*Homeopathic*	1 (0.4)	9 (0.8)	3 (2.2)	0 (0.0)	13 (0.8)	
**Non Pharmaceuticals**	**162 (68.4)**	**810 (67.9)**	**81 (58.3)**	**22 (52.4)**	**1075 (66.7)**	
*Industrial product*	44	272	40	7	363	
*Domestic product*	22	196	14	6	238	
*Cosmetic*	22	88	3	1	114	
*Caustic*	13	65	2	2	82	
*Plant*	27	50	1	0	78	
*Pesticide*	17	50	1	1	69	
*Food*	10	39	12	2	63	
*Other*	4	27	8	2	41	
*Substance abuse*	3	23	0	1	27	
**Route of exposition**						
Ingestion	160 (67.5)	961 (80.6)	100 (71.9)	33 (78.6)	1254 (77.8)	
Mucosal	45 (19.0)	137 (11.5)	18 (12.9)	1 (2.4)	201 (12.5)	
Cutaneous	11 (4.6)	38 (3.2)	11 (7.9)	4 (9.5)	64 (4.0)	
Inhalation	4 (1.7)	21 (1.8)	6 (4.3)	0 (0.0)	31 (1.9)	
Other	12 (5.0)	14 (1.2)	1 (0.7)	1 (2.4)	28 (1.7)	
Ocular	3 (1.3)	17 (1.4)	3 (2.2)	2 (4.8)	25 (1.6)	
Bite	1 (0.4)	5 (0.4)	0 (0.0)	0 (0.0)	6 (0.4)	
Injection	1 (0.4)	0 (0.0)	0 (0.0)	1 (2.4)	2 (0.1)	
**Clinical presentation**						
**Asymptomatic**	**224 (94.5)**	**1079 (80.4)**	**119 (85.6)**	**28 (66.7)**	**1450 (90.0)**	**0.002**
**Symptomatic**	**13 (5.5)**	**114 (9.6)**	**20 (14.4)**	**14 (33.3)**	**161 (10.0)**	
*Gastrointestinal*	4 (30.8)	76 (66.7)	10 (50.0)	4 (28.6)	94 (58.4)	
*Dermatological*	5 (38.5)	28 (24.6)	5 (25.0)	5 (35.7)	43 (26.7)	
*Neurological*	3 (23.1)	4 (3.5)	4 (20.0)	5 (35.7)	16 (9.9)	
*Other*	1 (7.7)	4 (3.5)	0 (0.0)	0 (0.0)	5 (3.1)	
*Respiratory*	0 (0.0)	2 (1.8)	0 (0.0)	0 (0.0)	2 (1.2)	
*Multi-systemic*	0 (0.0)	0 (0.0)	1 (5.0)	0 (0.0)	1 (0.6)	
**Emergency Department referral**						
No	**206 (86.9)**	**959 (80.4)**	**117 (84.2)**	**31 (73.8)**	**1313 (81.5)**	**0.048**
Yes	**31 (13.1)**	**234 (19.6)**	**22 (15.8)**	**11 (26.2)**	**298 (18.5)**	
**Decontamination**						
No	**222 (93.7)**	**1118 (93.7)**	**122 (87.8)**	**39 (92.9)**	**1501 (93.2)**	0.386
Yes	**15 (6.3)**	**75 (6.3)**	**17 (12.2)**	**3 (7.1)**	**110 (6.8)**	
*Charcoal*	6 (2.5)	43 (3.6)	9 (6.5)	3 (7.1)	61 (3.8)	
*Topic wash*	6 (2.5)	16 (1.3)	5 (3.6)	0 (0.0)	27 (1.7)	
*Ocular Irrigation*	3 (1.3)	15 (1.3)	3 (2.2)	0 (0.0)	21 (1.3)	
*Gastric lavage*	0 (0.0)	1 (0.1)	0 (0.0)	0 (0.0)	1 (0.1)	
***Total***	***237 (14.7)***	***1193 (74.1)***	***139 (8.6)***	***42 (2.6)***	***1611 (100.00)***	

*p-value* calculated for each variable

Industrial products (66.7%) were the most common non-pharmaceutical agents among all groups, followed by domestic and cosmetic products. Among pharmaceutical agents, the most frequent were analgesics (21.6%) and hormones (12.0%), significantly increasing with older age. 90% of children were asymptomatic, while gastrointestinal symptoms (58.4%) and dermatological symptoms (26.7%) were observed among symptomatic patients.

Table 2 shows the characteristics of children in Group P referred or not to the hospital by the PPCcP. Main reasons for admission were exposures to caustics, substances of abuse, plants, cardioactive, antihistaminics, neuroactives, analgesics or unclear parental communication,; adolescents and symptomatic children. Children referred to hospital had more outside exposure. Intentional exposure appeared more associated with hospital referral, while medication error was minor and mostly managed at home (90.1%). Ingestion was the most relevant route of exposure, and gastrointestinal symptoms the most relevant clinical presentation associated to Hospital referral.

**Table 2:** Risk of Hospital referral of “P” group, cases for whom PPCc was contacted by phone

Hospital Referral	Yes	Total	Univariable analysis		Multivariable logistic regression	
	n. (%)	n.	OR	95%CI	OR	95%CI
**Sex**						
Male	160 (19.3)	829	1.1	0.9–1.4		
Female	138 (17.6)	782	1.0			
*Total*	*298 (18.5)*	*1611*				
**Age category**						
Infant	31 (13.1)	237	1.0			
Pre School	234 (19.6)	1193	1.6*	1.1–2.4		
School	22 (15.8)	139	1.2	0.7–2.3		
Adolescent	11 (26.2)	42	2.4*	1.1–5.2		
*Total*	*298 (18.5)*	*1611*				
**Time of exposure**						
Morning	64 (17.3)	371	1.0		1.0	
Afternoon	123 (21.4)	575	1.3	0.9–1.8	1.5*	1.0–2.2
Evening	104 (16.6)	628	1.0	0.7–1.3	1.3	0.9–1.9
Night	7 (18.9)	37	1.1	0.5–2.7	1.2	0.5–3.3
*Total*	*298 (18.5)*	*1611*				
**Location of poison exposure**						
Outside	30 (42.3)	71	3.5*	2.1–5.7	3.9*	2.1–7.2
Home	268 (17.4)	1539	1.0		1.0	
*Total*	*298 (18.5)*	*1611*				
**Reasons for poison exposure**						
**Intentional exposure**	**7 (58)**	**12**				
*Abuse*	1 (100)	1				
*Suicide*	6 (54.5)	11	11.0*	2.7–44.4		
**Accidental exposure**	**291 (18)**	**1599**				
*General accidental exposure*	276 (19)	1452	2.1	1.2–3.8		
*Medication error*	14 (9.9)	142	1.0			
*Adverse reaction*	1 (20)	5	2.3	0.2–22.2		
*Total*	*298 (18.5)*	*1611*				
**Substance**						
Caustic	41 (50)	82	8.9*	3.3–23.9	44.8*	9.9–202.2
Substance of abuse	12 (44.4)	27	7.1*	2.2–23.2	22.0*	4.3–112.6
Other	16 (39.0)	41	5.7*	1.9–16.6	23.1*	4.6–114.8
Plant	25 (32.1)	78	4.2*	1.6–10.8	9.5*	2.1–44.1
Domestic product	37 (15.5)	238	1.6	0.7–3.9	8.0*	1.8–34.9
Industrial product	38 (10.5)	363	1.0	0.4–2.4	4.2	1.0–18.2
Pesticide	7 (10.1)	69	1.0		4.2	0.8–21.8
Cosmetic	12 (10.5)	114	1.0	0.4–2.8	4.3	0.9–20.4
Food	8 (12.7)	63	1.3	0.4–3.8	3.4*	0.7–17.3
Drug	102 (19)	536	2.1	0.9–4.7	1.0	
*Total*	*298 (18.5)*	*1611*				
**Pharmaceutical**						
Cardioactive	23 (53.59	43	29.9*	4.6–194.0	26.7*	5.6–128.0
Antistaminic	12 (42.9)	28	19.5*	3.1–123.9	17.4*	3.4–89.1
Neuroactive	18 (40)	45	17.3*	3.1–97.3	15.4*	3.2–73.4
Supplements	4 (16.7)	24	5.2	0.8–32.5	6.1*	1.0–37.2
Analgesic	20 (17.2)	116	5.4*	1.2–24.9	5.8*	1.3–26.4
None	196 (18.2)	1075	5.8*	1.4–24.1		
Homeopathic	2 (15.4)	13	4.7	0.6–39.4	5.8	0.7–46.4
Hormones	9 (13.8)	65	4.2	0.8–20.9	4.3	0.8–20.1
Other	2 (9.1)	22	2.6	0.3–20.2	3.4	0.4–26.7
Gastrointestinal	3 (13.6)	22	4.1	0.6–27.7	3.3	0.5–22.5
Topic	4 (7.4)	54	2.1	0.4–12.0	3.2	0.5–18.3
Respiratory	3 (6)	50	1.7	0.3–10.5	1.8	0.3–11.3
Antibiotic	2 (3.7)	54	1.0		1.0	
*Total*	*298 (18.5)*	*1611*				
**Route of exposition**						
Ingestion	271 (21.6)	1254	5.3*	2.7–10.2	5.8*	2.9–11.6
Eye	2 (8)	25	1.7	0.3–8.1	2.8	0.4–22.7
Other	2 (8.3)	24	1.7	0.4–8.5	2.4	0.5–12.7
Inhalation	4 (12.9)	31	2.8	0.8–9.8	2.7	0.7–10.7
Skin	8 (12.5)	64	2.7	1.0–7.3	1.5	0.5–4.7
Mucosa	10 (5)	201	1.0		1.0	
Injection	0 (0)	2	0.0			
Bite	0 (0)	6	0.0			
Missing	1 (25)	4	6.4	0.6–68.4	11.5	0.9–151.9
*Total*	*298 (18.5)*	*1611*	*1.7*			
**Clinical presentation**						
Gastrointestinal Symptoms	58 (61.7)	94	3.7*	1.7–8.4		
Neurologic Symptom	9 (56.3)	16	3.0	0.9–10.1		
Respiratory Symptom	2 (100)	2		0.0		
Others	2 (40)	5	1.5	0.2–10.6		
Dermatological Symptoms	13 (30.2)	43	1.0			
Multiple Symptoms	0 (0)	1	0.0			
*Total*	*84 (52.2)*	*161*				
**Decontamination**						
Charcoal	36 (59)	61	18.0*	3.1–103.8	10.0*	1.2–83.3
None	257 (17.1)	1501	2.6	0.6–11.0	2.1	0.3–16.0
Topic wash	2 (7.4)	27	1.0		1.9	0.2–23.7
Ocular irrigation	2 (9.5)	21	1.3	0.2–10.5	1.0	
Gastric lavage	1 (100)	1				
***Total***	***298 (18.5)***	***1611***				

OR with* are those with *p*-value < 0.05

### Group ED

Table 3 describes this population of 1075 children: 955 children directly acceded to the ED and 120 after a previous contact to our PPCcP (PED). Most children were males (54.6%), pre-school age (71.9%), afternoon and evening as times of exposure (77.9%), and home as location (92.0%).

**Table 3:** Characteristics of Group “ED”, cases accessing PPCc at Emergency Department

Age Category	Infant (n.95, 8.8%)	Pre-School (n.773, 71.9%)	School (n.99, 9.2%)	Adolescent (n.108, 10.0%)	Total	*p*-value
**Gender**						0.045
Male	56 (58.9)	423 (54.7)	61 (61.6)	47 (43.5)	587 (54.6)	
Female	39 (41.1)	350 (45.3)	38 (38.4)	61 (56.5)	488 (45.4)	
**Time of exposure**						< 0.001
Morning	19 (20.0)	148 (19.1)	19 (19.2)	9 (8.3)	195 (18.1)	
Afternoon	26 (27.4)	284 (36.7)	33 (33.3)	41 (38.0)	384 (35.7)	
Evening	43 (45.3)	325 (42.0)	41 (41.4)	45 (41.7)	454 (42.2)	
Night	7 (7.4)	16 (2.1)	6 (6.1)	13 (12.0)	42 (3.9)	
**Location of poison exposure**						< 0.001
Home	92 (96.8)	743 (96.1)	80 (80.8)	74 (68.5)	989 (92.0)	
Outside	3 (3.2)	30 (3.9)	19 (19.2)	34 (31.5)	86 (8.0)	
**Reasons for poison exposure**						< 0.001
**Accidental exposure**	**95 (100.0)**	**773 (100.0)**	**94 (95.0)**	**30 (27.8)**	**992 (92.3)**	
*General accidental exposure*	79 (83.2)	741 (95.9)	87 (87.9)	29 (26.9)	936 (87.1)	
*Medication error*	16 (16.8)	30 (3.9)	6 (6.1)	1 (0.9)	53 (4.9)	
*Abuse*	0 (0.0)	2 (0.3)	1 (1.0)	0 (0.0)	3 (0.3)	
**Intentional exposure**	**0 (0.0)**	**0 (0.0)**	**5 (5.0)**	**78 (72.3)**	**83 (7.7)**	
*Abuse substance*	0 (0.0)	0 (0.0)	1 (1.0)	41 (38.0)	42 (3.9)	
*Suicide attempt*	0 (0.0)	0 (0.0)	4 (4.0)	37 (34.3)	41 (3.8)	
**Categories of exposure**						
**Pharmaceutical**	**36 (37.9)**	**239 (30.9)**	**25 (25.3)**	**40 (37.0)**	**340 (31.6)**	**< 0.001**
Neuroactive	2 (2.1)	43 (5.6)	8 (8.1)	21 (19.4)	74 (6.9)	
Analgesic	8 (8.4)	42 (5.4)	6 (6.1)	12 (11.1)	68 (6.3)	
Cardioactive	5 (5.3)	47 (6.1)	2 (2.0)	3 (2.8)	57 (5.3)	
Hormones	4 (4.2)	33 (4.3)	0 (0.0)	0 (0.0)	37 (3.4)	
Antistaminic	2 (2.1)	16 (2.1)	2 (2.0)	0 (0.0)	20 (1.9)	
Respiratory	3 (3.2)	13 (1.7)	0 (0.0)	1 (0.9)	17 (1.6)	
Antibiotic	4 (4.2)	9 (1.2)	2 (2.0)	1 (0.9)	16 (1.5)	
Topic	5 (5.3)	10 (1.3)	0 (0.0)	0 (0.0)	15 (1.4)	
Other	1 (1.1)	8 (1.0)	3 (3.0)	2 (1.9)	14 (1.3)	
Supplements	1 (1.1)	9 (1.2)	1 (1.0)	0 (0,0)	11 (1.0)	
Gastrointestinal	1 (1.1)	4 (0.5)	1 (1.0)	0 (0.0)	6 (0.6)	
Homeopathic	0 (0.0)	5 (0.6)	0 (0.0)	0 (0.0)	5 (0.5)	
**Non Pharmaceutical**	**59 (62.1)**	**534 (69.1)**	**74 (74.7)**	**68 (63.0)**	**735 (68.4)**	**< 0.001**
Domestic product	12 (12.6)	173 (22.4)	18 (18.2)	5 (4.6)	208 (19.3)	
Industrial product	17 (17.9)	138 (17.9)	23 (23.2)	4 (3.7)	182 (16.9)	
Caustic	4 (4.2)	118 (15.3)	11 (11.1)	9 (8.3)	142 (13.2)	
Abuse Substance	4 (4.2)	13 (1.7)	1 (1.0)	41 (38.0)	59 (5.5)	
Other	9 (9.5)	18 (2.3)	13 (13.1)	4 (3.7)	44 (4.1)	
Plant	4 (4.2)	27 (3.5)	0 (0.0)	0 (0.0)	31 (2.9)	
Cosmetic	2 (2.1)	26 (3.4)	1 (1.0)	1 (0.9)	30 (2.8)	
Food	4 (4.2)	10 (1.3)	5 (5.1)	4 (3.7)	23 (2.1)	
Pesticide	3 (3.2)	11 (1.4)	2 (2.0)	0 (0.0)	16 (1.5)	
**Route of exposition**						< 0.001
Ingestion	77 (81.1)	707 (91.5)	79 (79.8)	92 (85.2)	955 (88.8)	
Cutaneous	3 (3.2)	27 (3.5)	8 (8.1)	2 (1.9)	40 (3.7)	
Inhalation	6 (6.3)	3 (0.4)	6 (6.1)	14 (13.0)	29 (2.7)	
Mucosal contact	6 (6.39	19 (2.5)	2 (2.0)	0 (0.0)	27 (2.5)	
Ocular	0 (0.0)	12 (1.6)	4 (4.09	0 (0.0)	16 (1.5)	
Other	3 (3.2)	4 (0.5)	0 (0.0)	0 (0.0)	7 (0.7)	
Injection	0 (0.0)	1 (0.1)	0 (0.0)	0 (0.0)	1 (0.1)	
**Clinical presentation**						< 0.001
**Asymptomatic**	**80 (84.2)**	**584 (75.6)**	**55 (55.6)**	**33 (31.6)**	**752 (70.0)**	
**Symptomatic**	**15 (15.8)**	**189 (24.4)**	**44 (44.4)**	**75 (69.4)**	**323 (30.0)**	
*Gastrointestinal*	8 (53.3)	130 (68.8)	23 (52.3)	18 (24.0)	179 (55.4)	
*Neurologic*	5 (33.3)	23 (12.2)	5 (11.4)	44 (58.7)	77 (23.8)	
*Dermatological*	1 (6.7)	24 (12.7)	12 (27.3)	3 (4.0)	40 (12.4)	
*Multiple*	1 (6.7)	3 (1.6)	1 (2.3)	4 (5.3)	9 (2.8)	
*Respiratory*	0 (0.0)	3 (1.6)	3 (6.8)	3 (4.0)	9 (2.8)	
*Others*	0 (0.0)	6 (3.2)	0 (0.0)	3 (4.09	9 (2.8)	
**Hospital admission**						< 0.001
No	53 (55.8)	433 (56.0)	49 (49.5)	29 (26.9)	564 (52.5)	
Yes	42 (44.2)	340 (44.0)	50 (50.5)	79 (73.1)	511 (47.5)	
**Decontamination**						< 0.001
None	83 (87.4)	632 (81.8)	84 (84.8)	81 (75.0)	880 (81.9)	
Charcoal	9 (9.5)	106 (13.7)	9 (9.1)	17 (15.7)	141 (13.1)	
Gastric lavage & charcoal	1 (1.1)	17 (2.2)	1 (1.0)	6 (5.6)	25 (2.3)	
Ocular irrigation	0 (0.0)	14 (1.8)	4 (4.0)	0 (0.0)	18 (1.7)	
Topic wash	1 (1.1)	3 (0.4)	1 (1.0)	0 (0.0)	5 (0.5)	
Gastric lavage	0 (0.0)	1 (0.1)	0 (0.0)	4 (3.7)	5 (0.5)	
Intestinal irrigation	1 (1.1)	0 (0.0)	0 (0.0)	0 (0.0)	1 (0.1)	

*p-value* calculated for each variable

In the infant group, 96.8% were exposed at home and 44.2% admitted to the hospital. The pre-school group was exposed to domestic products (22.4%), while the School group was exposed with higher frequency to industrial products (, to neuroactive and analgesics drugs (6.1%). The adolescent group showed: intentional exposure (72.3%) female gender (56.5%) and home intake (68.5%), with exposure to neuroactive (19.4%), analgesics (11.1%) and cardioactive drugs (2.8%) and caustic products (9.0%). The most prevalent substance abuse was alcohol (50.0%), followed by Cannabis (30.0%).

Table 4 shows frequencies of children in Group ED. Night appears to be a time for exposure. Children with outside exposure had a greater likelihood to be admitted to the hospital had an outdoor exposure, with no gender difference. Approximately 85% had a hospital stay of less than two days in hospital, while for the 15% of cases hospitalization lasted longer. This latter group included adolescents with intentional exposure and neurological symptoms, hospitalised after a first decontamination procedure. Regarding suicide attempts, A total of 41 children were admitted after suicide attempt: 21 patients to the psychiatric ward, 9 in the paediatric ward, 5 patients required surgery due to caustic exposure and 6 cases required PICU admission.

**Table 4:** Risk of Hospital admission of Group “ED”, cases accessing PPCc at Emergency Department

Hospital admitted	Yes	Total	Univariable analysis			Multivariable logistic regression	
	n(%)	n(%)	OR	95%CI		OR	95% CI
**Sex**							
Female	245 (50.2)	488	1.2				
Male	266 (45.3)	587	1.0	1.0–1.5			
*Total*	*511 (47.5)*	*1075*					
**Age category**							
Infant	42 (44.2)	95	1.00	0.7–1.6			
Pre School	340 (44)	773	1.00				
School	50 (50.5)	99	1.29	0.9–2.0			
Adolescent	79 (73.1)	108	3.46*	2.2–5.5			
*Total*	*511 (47.5)*	*1075*					
**Time of exposure**							
Morning	77 (39.5)	195	1.0			1.0	
Afternoon	188 (49)	384	1.5*	1.0–2.1		1.5*	1.0–2.2
Evening	215 (47.4)	454	1.4	1.0–1.9		1.4	1.0–2.0
Night	31 (73.8)	42	4.3*	2.0–9.4		3.2*	1.4–7.0
*Total*	*511 (47.5)*	*1075*					
**Location of poison exposure**							
Outside	57 (66.3)	86	2.3*	1.45–3.7			
Home	454 (45.9)	989	1.0				
*Total*	*511 (47.5)*	*1075*					
**Reasons for poison exposure**							
**Intentional exposure**	**71 (84.5)**	**84**					
*Suicide attempt*	41 (97.6)	42	86.82*	5.3–1400			
*Abuse substance*	30 (71.4)	42	5.29*	2.0–13.9			
**Accidental exposure**	**440 (44.4)**	**991**					
*General accidental exposure*	420 (44.9)	935	1.72	1.0–3.1			
*Medication error*	17 (32.1)	53	1.00				
*Adverse reaction*	3 (100)	3	5.29*				
*Total*	*511 (47.5)*	*1075*					
**Substance**							
Substance of abuse	43 (72.9)	59	17.5*	4.1–75.2		15.9*	4.7–53.1
Caustic	100 (70.4)	142	15.5*	4.5–53.7		15.6*	5.1–47.6
Other	29 (65.9)	44	12.6*	3.0–53.1		11.7*	3.4–40.0
Food	10 (43.5)	23	5.0*	1.2–21.0		5.0*	1.3–19.0
Domestic product	80 (38.5)	208	4.1*	1.3–12.3		4.1*	1.4–12.3
Plant	11 (35.5)	31	3.6	0.9–13.6		3.7*	1.0–13.3
Industrial product	53 (29.1)	182	2.7	0.9–8.1		2.7	0.9–8.2
Drug	178 (52.4)	340	7.1*	2.4–21.5		2.2	0.5–10.4
Pesticide	3 (18.8)	16	1.5	0.3–7.9		1.7	0.3–8.6
Cosmetic	4 (13.3)	30	1.0			1.0	
*Total*	*511 (47.5)*	*1075*					
**Pharmaceutical**							
Neuroactive	55 (74.3)	74	8.7*	2.2–34.0		8.7*	2.5–30.2
Antistaminic	13 (65)	20	5.6*	1.1–27.9		5.5*	1.3–23.9
Other	9 (64.3)	14	5.4	0.9–30.8		5.3*	1.1–25.6
Cardioactive	35 (61.4)	57	4.8*	1.3–17.9		4.9*	1.4–17.1
Analgesic	33 (48,5)	68	2.8	0.8–9.9		2.8	0.8–9.4
Homeopathic	2 (40)	5	2.0	0.2–17.9		2.0	0.2–16.9
Gastrointestinal	2 (33.3)	6	1.5	0.2–12.2		1.6	0.2–12.6
Supplements	4 (36.4)	11	1.7	0.3–9.5		1.5	0.3–8.1
Respiratory	6 (35.3)	17	1.6	0.4–7.6		1.5	0.3–7.0
Hormones	11 (29.7)	37	1.3	0.3–4.9		1.3	0.3–4.8
Topic	4 (26.7)	15	1.1	0.2–5.6		1.1	0.2–5.4
Antibiotic	4 (25)	16	1.0			1.0	
None	333 (45.3)	735	2.5	0.8–7.8			
*Total*	*511 (47.5)*	*1075*					
**Route of exposition**							
Inhalation	19 (65.5)	29	8.2*	1.6–43.4			
Ingestion	464 (48.6)	955	4.1*	1.2–14.5			
Cutaneous	15 (37.5)	40	2.6	0.6–11.0			
Other	2 (28.6)	7	1.7	0.2–14.5			
Mucosa	7 (25.9)	27	1.5	0.3–7.1			
Ocular	3 (18.8)	16	1.0				
Injection	1 (100)	1					
*Total*	*511 (47.5)*	*1075*					
**Clinical presentation**							
Neurologic Symptom	62 (80.5)	77	5.6*	2.2–14.0			
Gastrointestinal Symptoms	137 (76.5)	179	4.4*	2.1–9.3			
Respiratory Symptom	6 (66.7)	9	2.7	0.6–12.9			
Others	5 (55.6)	9	1.7	0.4–7.4			
Multiple Symptom	4 (44.4)	9	1.1	0.2–4.7			
Dermatological Symptoms	17 (42.5)	40	1.0				
*Total*	*231 (71.5)*	*323*					
**Decontamination**							
Gastric lavage & carbon	22 (88)	25	29.3*	1.2–703.8			
Charcoal	100 (70.9)	141	9.8*	1.0–94.9			
None	378 (43)	880	3.01	0.3–27-1			
Ocular irrigation	4 (22.2)	18	1.1	0.1–14.1			
Topic wash	1 (20)	5	1.0	.			
Gastric lavage	5 (100)	5		.			
Intestinal Irrigation	1 (100)	1					
***Total***	***511 (47.5)***	***1075***					

OR with* are those with *p*-value <0.05

PICU admission was necessary for 41 of the hospitalized patients (8%). Causes for admission were: 34.1% drugs, 24.4% caustics, 17.1% substances of abuse, 14.6% viper bites, 4.9% boric acid, 2.4%, botulinum and 2.4% disk battery ingestion. Only 30% required intensive treatment (mechanical ventilation, dialysis, endoscopy, drug support treatment, etc.) while 70% needed observation and advanced monitoring.

## Discussion

Poisoning is a common and potentially life-threatening clinical condition for children and exposure to xenobiotics, even mild, is a frightening event for parents and an important reason for referral to the ED**.** The aim of this study was to describe and analyse features of children exposed to xenobiotics. We analyzed data collected by our PPCc over a three-year period. To our knowledge, this is the only prospective study presenting exclusively paediatric data and stratified by age groups, each with their own characteristics.

In Europe, under ECHA (European Chemical Agency) supervision, European Union Countries have their own National Poison Centres for giving information and collecting data on hazardous substances [2–5]. Our centre is one of Italian Poison Control Centres and the one dedicated exclusively tochildren and managed, in the three years of activity here reported, 2566 children, aged 0–18, divided in two groups: *P* (children whose parents or caregivers contacted by phone the PPCc) and *ED* (children directly acceding to our Hospital). A total of 1611 phone calls were registered, of which 1313 were managed by phone counselling alone, without need to ED referral. This unique telemedicine service has a pivotal role, since it provides specialized support, avoiding unnecessary accesses to the hospital, with flux reduction in ED, decreasing both individual and collective risk of infectious disease spread. This last risk has been particularly relevant in the current Covid-19 pandemic reducing also health care costs for the population [6, 7]. The description of services provided by PPCcP and PPCcED accesses is also useful to public health authorities in order to describe the risk of xenobiotic exposure in children and adolescents and to identify prevention measures and policies to be implemented.

Pre-school was the age group most frequently managed by PPCc, reflecting the unintentional exposure due to environment exploring attitudes typical of this age. Accidental exposure to pharmaceuticals appeared an important cause of exposure in all ages among P and ED groups. The most frequent drugs exposure in infants, pre-school and school age children involved analgesics, a category of drugs often available at home, while for the adolescent group is represented by neuroactive drugs, often taken intentionally by this age group.

Data for the age group 14–18 years could be underestimated as these patients can also be referred to an adult ED. Nevertheless, our PPCcP plays an important role within local health reality and especially for this age group, it represents a guide for other hospitals. This aspect underlines the importance of a specific pediatric poison control center able to carry out a correct management of xenobiotic intoxication in all pediatric ages.

Among the younger age groups, the leading categories of exposure were drugs, industrial and domestic products, followed by cosmetics, caustics, plants and pesticides. Interestingly, plantexposure is a frequent reason of PPCcP consultation among youngsters probably due to the overestimation of the danger and also because plants and their toxicity represent an unknown world [8]. This huge variety of xenobiotics reflects the heterogenous types of products available around children’s world. Being surrounded by such a large amount of products represents a potential hazard for children. This is a reason why exposure to xenobiotics happened most frequently via the oral route, because of the acquired competence of this age group*.*

Medication errors represented an uncommon exposure and our experience shows that most of them were managed at home (90.1%) and no referral to hospital was necessary. However, this phenomenon deserves particular attention because it is not determined directly by the child. Data collected suggested that exposures are due to difficult management and to limited experience in drug administration. Even drug labelling and packaging complexity may increase difficulties in finding and understanding information. Similarly, socioeconomic status and poor awareness of side effects can play an important role. Although linguistic difficulties in the medical prescription interpretation could be considered as the main reason for an erroneous drug administration, however, in our case series most of these errors were made by Italian and non-foreign parents. Moreover, some parents may also intentionally give children additional doses, if the prescribed doses do not achieve the desired outcome (e.g. antipyretics and cold medicines) increasing the risk of side effects [9]. Around 36–67% of the drugs used in paediatrics age are drugs administered off-label [10], and this aspect leads more frequently to medication errors [11, 12]. Off label drug use in children requires special attention and detailed instruction that are not always reported in the leaflets and need adjunctive instructions provided by the prescribing physician. Furthermore, safety data relating to the use of medicines in children are limited and not always possible to extrapolate them from the information available on adult studies [11, 12]. In our experience, adverse drug reactions represent a rare occurrence and the small number of cases mirrors the trend reported in literature [13]. However, to our knowledge, adverse drug reactions are hardly described by the PPCcP also because this event is directly referred to ED. Since PPCcP physicians are competent also to manage ADR, an educational action should be directed to the population, in order to refer more frequently to PPCcP and consequently to reduce inappropriate access to ED.

We reported a high percentage of asymptomatic cases (90%), higher in infant, pre-school and school groups, probably due to parental anxiety that inversely correlates with aging. Hospital referral was recommended for 18.5% of cases calling the PPCcP, with higher prevalence for pre-school and adolescent age group. A limitation of this study is that it was not possible to follow up patients who were referred to other hospitals after having a contact with the PPCcP.

Suicide attempts referred to ED are infrequent but remain a cause of concern, in fact suicide is the second leading cause of death in adolescents [14]. Generally, suicide attempts are numerically higher than actual suicides, but the real number is underestimated because it is difficult to obtain reliable information. We found a low number of consultations to the PPCcP all performed by the parents of asymptomatic older teenagers, with a tendency of downplaying what happened, while those directly referred to ED were symptomatic with mild/severe presentation and in a greater number. We believe that these types of exposures rarely makes little use of PPCcP. A significant association was not detected between age and severity of intoxication. Suicide attempts can be a risk factor for suicidal behaviour. Although in Italy suicide rates are the lowest in Europe, the presence of the risk factors listed in literature must be strictly evaluated and actively treated to avoid suicides [15, 16]. In our study, ingestion results the principal route of exposure. Enteric decontamination (DE) prevents the absorption of xenobiotics from the gastrointestinal system and includes the use of activated charcoal, gastric lavage, cathartics, whole bowel irrigation and ipecac or other emetics. To our knowledge, in the paediatric age group, activated charcoal and gastric lavage remain the most frequently treatments. In our study, 18,1% of our patients received a DE treatment. In children the use of activated charcoal or gastric lavage depends on the type of xenobiotic, modality exposure and time of latency. However, it is necessary to consider that invasive procedures are more difficult to perform and present major challenges, which may worsen the outcome. Consensus of clinical toxicologists showed that activated charcoal in the case of drug poisoning may prevent absorption if given within an hour [17, 18].

In about 8% of cases, admission to the PICU was required. Approximately 70% of hospitalizations in the PICU required only advanced vital signs monitoring, and this is similar to what has been reported in literature [19, 20]. The reason is that in most cases, after an exposure, they may not show symptoms when arriving to the ED. In these cases, if the exposure is confirmed by the clinical history and the xenobiotic is dangerous, it is reasonable to think that the maximum absorption of the xenobiotic has not been achieved yet and PICU admission may be therefore advised. In our study, fatality is an event that did not occur, in accordance with current literature [21–23]. Differently from adults, the fatality rate of paediatric poisoning is much lower because most of them are unintentional and the ingested xenobiotic dose is too low to induce severe intoxication.

## Conclusions

The results of this study describe in detail the types of xenobiotics most frequently involved in paediatric exposures, requiring hospitalization and, they show the role of PPCc, both by Phone and directaccess to the ED, as the figure that can manage all of the cases. As a consequence of PPCcP, a larger number of cases can be initially screened and remotely managed, avoiding unnecessary hospital referral, Our experience confirms that mortality is low in paediatric age and most cases of toxic exposures are either asymptomatic or develop mild symptoms. This information represents the foundation for avoiding unnecessary medicalization and hospitalization. The data collected also indicate that intentional exposure is significant in the adolescent group, where there is an autonomous use of the substances. Furthermore, as underlined by World Health Organization, suicide attempts or self-harm in adolescents are an emerging health problem worldwide and poisoning with drugs represents the most used method. In our opinion an exclusively pediatric Poison Control Center can represent the opportunity for not only a better care, but also for an improved understanding of pediatric and providing useful information and implementing successful preventive strategies.

## Data Availability

The datasets used and/or analysed during the current study are available from the corresponding author on reasonable request.
